# Australian Generation Z and the Nexus between Climate Change and Alternative Proteins

**DOI:** 10.3390/ani12192512

**Published:** 2022-09-21

**Authors:** Diana Bogueva, Dora Marinova

**Affiliations:** 1 Curtin University Sustainability Policy (CUSP) Institute, Curtin University, GPO Box U1987, Perth, WA 6845, Australia; 2 Centre for Advanced Food Engineering (CAFE), University of Sydney, Sydney, NSW 2006, Australia

**Keywords:** Generation Z, alternative proteins, climate change, sustainable food, cultured meat, insect-based food, plant-based meat, sustainability, protein transition

## Abstract

**Simple Summary:**

Existing food production systems and dietary behaviours are not healthy nor sustainable due to the higher environmental footprint of animal-derived foods. Alternative dietary choices could provide better outcomes. We investigated Generation Z (Gen Z)’s alternative food preferences within a planetary health framework which combines human and ecological well-being. The paper is based on data from a 2021 cross-national survey conducted in the main Australian cities, which explored Gen Z’s attitudes towards meat consumption in relation to climate change and alternatives to animal-based proteins. Climate change is seen as a result of human activities by 86% of the survey participants. More than a third (38%) of them believe that livestock production and the consumption of animal-sourced foods are contributing significantly to climate change and environmental deterioration. The remaining majority (62%), however, has a low awareness and understanding of the food systems’ impacts, disbelieving that diet is a major contributor to climate change. We discuss how these findings will shape Australia’s Gen Z, climate change and the future of alternative proteins.

**Abstract:**

Scientific evidence shows that current food systems are impacting the planet in ways that are unsustainable and detrimental to human health. Various technological advances have been made in response, one of them being the development of new food products known as novel alternative proteins, including cultured meat, plant-based meat analogues, algae- and insect-based foods. The future of these alternative proteins to a large extent depends on consumer acceptance from young people. This study investigates the attitudes of Australia’s adult Generation Z (Gen Z), born between 1995 and 2003, regarding climate change and more sustainable food choices. Gen Z is a diverse, important and trendsetting group known for organising globally on causes related to climate, social justice and health. The study of Australia’s Gen Z is based on a 2021 cross-national survey in the main Australian cities. It shows that, although 86% of the participants perceive climate change as anthropogenic, only 38% believe that livestock-based foods are contributing significantly to global warming and environmental deterioration. The paper discusses the implications for Gen Z and novel alternative proteins given that the majority of Australia’s young people has low awareness of the environmental impacts of food systems and dietary choices.

## 1. Introduction

Transitioning to sustainability requires people across the globe to make environmentally better food choices. Technology and efficiency improvements also play a critically important role in responding to climate change and dealing with other environmental challenges. For the global human population, food security is a major problem exacerbated by ecological and humanitarian crises as well as by everyday consumer behaviour. This study specifically looks at Generation Z (Gen Z) consumers and gauges their attitudes towards climate change and food choices that reduce the human impact on the environment.

### 1.1. Future Food Security

According to the United Nations’(UN) forecasts, the global human population is expected to exceed nine billion by mid-century and will need around 70% more food to feed everyone [1]. This has to happen without irreparably harming the natural environment and preferably without or with very limited expansion of the land area already used for agriculture [2]. Food demand for the growing global population will intensify significantly the already intense competition over access to the Earth’s limited natural resources and will put even more pressure on the planet’s fragile ecosystems. Any substantial transition to a just and sustainable world will need to involve the global consumer shifting away from food consumption lifestyles that rely on products with a large environmental footprint, such as red meat. This is particularly true for high-income countries where meat consumption is high and continues to increase [3]. 

Despite consumers becoming increasingly aware in recent years of the climate costs of their actions and choices, some contributing factors such as diet are still not on their radar. Current food production systems and dietary behaviours are neither healthy nor sustainable. Poor diets are responsible for more of the global burden of diseases than sex, drugs, alcohol and tobacco combined [2]. Animal-derived foods, particularly red and processed meat, have the largest impact on the natural environment in terms of land use, soil degradation, greenhouse gas emissions, terrestrial biodiversity loss, and can also contribute to the development of non-communicable diseases, such as cancer, diabetes and cardiovascular disease [2]. 

Current food systems represent an obvious problem that people are largely unaware of and those who are reducing their intake of animal-based proteins because of environmental concerns are a small minority [4]. Discussions about the serious impacts of food are also largely absent from high-level international sustainability forums, such as the meetings of the Conference of the Parties (COP) to the UN Framework Convention on Climate Change (UNFCCC). The 2021 UN COP26 in Glasgow was a lost opportunity for urgent coordinated action regarding food systems but there are calls now to include food-related emissions in the national climate plans for COP27 [5]. In addition to burning fossil fuels, deforestation, land degradation, misappropriation of water and other resources, application of chemical fertilisers and the threat of zoonotic diseases are all issues associated with the consumption of livestock products by wealthier sections of society. They impact on current and future food security and any change towards more sustainable dietary options matters. 

### 1.2. Why Does It Matter? 

The global food system is currently responsible for about a quarter of all human-made greenhouse gases [6], a figure that is projected to increase. The increase in food systems emissions alone threatens global warming of above 1.5 °C [7,8]. The Special Report of the Intergovernmental Panel on Climate Change (IPCC) exposes that between 10.8 and 19.1 billion tonnes of CO_2_-equivalent emissions per year are coming from food [9,10]. This represents between 21% and 37% of global total emissions with the difference of 16% based on how parameters such as land use, agricultural production, supply chains and post- retail operations are included in the way food emissions are calculated. Contributing factors such as consumer food preparation, cooking and consumer waste also need to be included [11] making the greenhouse gas reduction challenge even harder. 

In an effort to solve these pressing future problems, technologists and scientists are using a variety of new biotechnological approaches and the fundamental principles of chemistry, physics, biology and engineering to create a new generation of foodstuff known as novel alternative proteins. These new alternatives, including plant-based meat analogues, cultured meat, fermented food, algae and insect-based foods, are gaining momentum and creating new opportunities in the protein production systems [12]. Some see them as disruptors of the current agriculture and food industry, particularly the multibillion-dollar global meat industry [13]. For example, it is projected that by 2040 only 40% of the global meat consumption will still come from conventional sources, such as livestock [13]. 

With the protein alternatives coming to the market, the most important factor for their success (or failure) would be what consumers think [13], particularly younger people who will shape any future food trends [14,15,16,17,18,19,20]. Gen Z (born between 1995 and 2010) is the largest population cohort in human history. It represents 30% of the world’s future buying and decision-making power. This generation is tech-savvy [21], educated, community oriented, environmentally and sustainable living conscious [22], drives social change through initiatives such as School Strike 4 Climate and Black Lives Matter and is an increasingly trendsetting influential group [23]. In Australia, it represents 20% of the total population and its members are already aware of the sustainability challenges they will face in the near future [12]. Are alternative proteins part of Gen Z’s pathways to sustainability? 

This study explores Australian Gen Z, seeking to understand what young people think, want, need and expect from the climate change and alternative proteins nexus. After describing the methodology of the study, a demographic overview of the Gen Z participant sample is provided followed by the survey results related to its perceptions about the main contributors to climate change. The nexus between human food choices and climate change is then discussed using qualitative excerpts from the Gen Z survey. Australian Gen Z’s thoughts and expectations related to novel alternative proteins are then conveyed. The paper concludes with reflections about the stance Gen Z is taking towards this new category of foods. 

## 2. Methodology

The methodology employed in the study of Australia’s Gen Z is based on a mixed-methods approach which combines some quantitative descriptions with qualitative analysis within a single survey [24]. It is based on the assumption that a mix of positivist, fact-based findings with qualitative subjective interpretations and personal attitudes can provide a better understanding of the nexus between climate change and food choices [25]. Methodologically, this approach sits between the philosophical paradigms of interpretivism and pragmatism [26], which aligns well with the research aim to provide an understanding about Gen Z’s attitudes towards the new class of foods described as alternative proteins. The single-survey design also allows us to bridge the gap between what are typically deductive quantitative analysis and reflexive qualitative data interpretation by reflecting on quantitative evidence and deducting from qualitative statements [27]. 

A single questionnaire combining multiple-choice and open-ended questions was used to gauge Australian Gen Z’s attitudes. It was designed for adult representatives of Gen Z aged between 18 and 26 years of age. The first section of the questionnaire was constructed to determine the demographic profile of the participants. Participants’ opinion regarding the major causes of climate change and the degree of their environmental awareness were covered in the second part of the questionnaire. They were given a list of options for the factors they considered to have a major influence on climate change, namely agriculture, consumption practices, deforestation and biodiversity loss, fast fashion, fossil fuels, industry, political inaction, population growth, transport and waste, plus an open-ended “other” category. Multiple choices were allowed but there was no comparison or ranking of the factors influencing climate change. A particular definition of “major” was not provided, as the aim was to capture the participants’ general understanding of what is important in dealing with climate change and their awareness of key factors that contribute to global warming. The participants were also asked directly whether people’s food choices are a major contributor to climate change with a “yes”/”no” answer and space to elaborate why they were of that opinion. This aimed at allowing the participants to share their thoughts about food’s impact on climate change. The third part elicited information on Gen Z’s perceptions of and attitudes towards alternative proteins and their attributes, associated with being more sustainable, as well as views about future perspectives for these novel foods. A question asked the participants specifically whether they accepted cultured (also referred to as lab-grown, cell-based, in-vitro or clean) meat as a new alternative protein with open-ended space provided to explain why they were of such an opinion. Those who rejected cultured meat were asked about their willingness to consume other alternatives to animal meat, such as insects, algae, new plant-based alternatives and traditional fruit and vegetables. Prior to being administered, the survey was pilot tested with a sample from the targeted population to identify whether sufficient response categories were available and whether there were problems with any of the questions.

The collected data was investigated for basic statistical distributions and thematically in order to elicit explanations about the opinions voiced by Gen Z within a nutrition/protein transition framework. To facilitate this, the themes were also analysed with NVivo 12 software. 

This study has been approved by the Curtin University Human Research Ethics Committee.

## 3. Findings and Discussion

### 3.1. Demographic Description of the Sample

In 2021, an online survey was conducted across Australia’s major cities of Sydney, Melbourne, Brisbane, Perth, Canberra and Adelaide. The actual study participants were recruited using simple random sampling (based on computer-generated random numbers) from a database of 35,000 people who have previously indicated willingness to participate in surveys. For each city, the target was to obtain more than 100 participants. Each individual in the sample frame for every city was assigned a unique computer-generated number guaranteeing that everyone in the respective population had the same probability of being selected. This random selection decreased the chance of bias. In total, there were 700 invitations sent to the participants of the random sample two of which bounced back. Out of 698 randomly invited participants, 478 responded by completing the survey, generating a response rate of 68.5%. The obtained sample is statistically representative of Australia’s Gen Z with 95% confidence level and confidence interval of 4.48. All participants gave electronic consent for participation in the survey (by ticking the “yes” box).

There was almost equal gender representation—49.8% or 238 male and 50.2% or 240 female respondents. The various age years between 18 and 26 were also almost evenly represented (see Table 1), with 51 participants aged 18, 55 aged 23 and the remaining ages with 52 to 54 participants. Given that the sample comprised young people, the majority (86%) were single, 14% married or in a de facto relationship; 92% had no children, 5% had one and 3% two children. The majority of participants (63%) belonged to middle-income households between A$51,000 and $100,000 per annum with relatively equal share of lower- (18%) and higher- (19%) income households (see Table 1). 

One in five participants was studying full-time, 41% were only working and 39% were combining work and study (see Table 1). By comparison with Australia’s population within this age bracket [28], this sample had a much higher share of people studying—20% (compared to around 8%), and 0% unemployed (compared to around 17%). This means that the participants had a relatively higher level of education and were more engaged with the Australian labour market. Their opinions are expected to be better informed than the rest of the Australian population and more attuned to the current challenges faced in Australia and globally. In other words, the insights obtained from this sample are more cognisant and likely to be more up to date with the latest developments in the climate change and alternative proteins nexus.

### 3.2. Australia’s Gen Z and Climate Change

**Figure 1:**
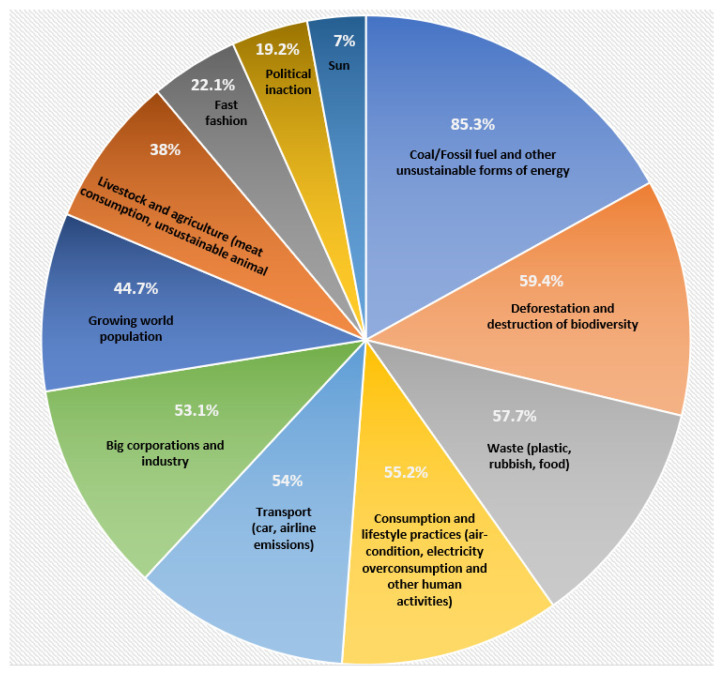
Main contributors to climate change (percentage of total sample) according to the study’s sample of Australian Gen Z.

When asked about the main contributors to climate change and presented with a long list of factors allowing multiple choices and an open-ended “Other” option to include another opinion, the majority of the participants, 85% (n = 408), answered with coal, fossil fuels and other unsustainable forms of energy (see Figure 1). The results categorically showed Gen Z’s awareness of the need for a low-carbon future away from coal and other fossil fuels. Representing the lifeblood of today’s modern economy, fossil fuels have powered the industrial revolution, but are now set for a transition towards decarbonisation. Gen Z is igniting such a change by putting pressure on policy makers with initiatives, such as climate strikes and #FridaysForFuture protests, which mobilised more than 1.6 million people around the globe [12,29,30,31]. 

The second largest contributor to climate change identified by the survey participants was deforestation and biodiversity loss (59% or n = 284), representing Gen Z’s knowledge and awareness. These young people’s understanding that habitat loss results in reduced biodiversity and contributes towards climate change is similarly expressed in initiatives, such as the Global Youth Biodiversity Network [32]. In the “Other” option, some participants added texts that also relate to this factor, for example, “destruction of nature”, “forest ecosystem loss” and “extinction of plant and animal species”. One participant wrote: “I think forest ecosystem loss is a big threat… We under-appreciate that nature is quite an important topic, but not many people my age care much about it. Perhaps we should do something about influencing the government to take actions” (born 1998, female, from Adelaide). Gen Z is also aware of its future responsibilities. Another participant described this: “Unfortunately expanding agriculture, residential and industrial development are causing loss of nature. It will be up to each of us to look after nature, to stop species become extinct, to stop climate change, but we must do it together” (born 2000, male, from Sydney).

The third contributing factor identified based on its importance was waste—plastic, rubbish and food waste (58% or n = 276). Gen Z recognises plastics as being among the most persistent pollutants on the planet contributing to greenhouse gas emissions and climate change [33]. Globally, wasted food accounts for about 8% of all greenhouse gas emissions [34]. Reducing food waste and the large environmental costs of producing food that is never consumed are a serious situation recognised by Gen Z. 

Consumption and lifestyle practices, such as use of air-conditioning, overconsumption of electricity and other human activities, were listed by 55% (n = 264) of the participants, followed by transport (54% or n = 259) and large industry (53% or n = 254). These practices inevitably lead to creating environmental pressures identified by Gen Z as problematic. The use of convenience products and services as part of people’s adopted lifestyle, such as driving a car, heating the house with fossil fuels or flying for leisure contribute to large carbon footprints. According to a survey conducted by Deloitte, the majority of Gen Z is making some attempts to reduce its environmental impact with 64% willing to pay more for an environmentally sustainable product (versus 36% who would go for a cheaper unsustainable product) [35]. The results from Australia’s Gen Z indicate a relatively good level of understanding of the impact energy, waste, consumption, transport and industry have on climate change with the majority of young people recognising these to be a problem. A Pew Research Centre [36] study shows American Gen Z in particular being active around climate change. 

Global population growth was seen as a factor for climate change by 45% (n = 214) of Gen Z. Many young people around the world are making a deliberate decision to have fewer children in order to reduce carbon emissions and the pressure on the ecological environment [37]. The complexity of population growth as it intertwines with consumption patterns, however, was outside the scope of this study.

Livestock and agriculture, including meat consumption and unsustainable animal farming practices, was included in the list of possible contributors to climate change, but only 38% (n = 182) indicated this to be a factor (see Figure 1). This is contrary to the existing evidence about the contribution of livestock and unsustainable animal raising practices [2,11,38]. A report by the World Economic Forum [39] analysed supply chains across the world responsible for more than half of the global emissions and identified food to be the main one, contributing more than 25%. Again, the level of awareness by Australia’s Gen Z appears to be inadequate. In the open-ended questions, many participants rather spontaneously raised concerns about animal welfare which appeared to be more prominent than direct association with climate change. Gen Z saw such ethical issues at the core of vegetarian and vegan food options. Similar conclusions around ethically-driven choices were stated in a UK-based study of Gen Z [14]. Animal welfare is a justified concern with climate change as livestock is increasingly exposed to heat waves, droughts, floods and feed shortages (e.g., [40]); however, it was surprising that this agricultural sector’s contribution to global warming was not recognised by 62% of the participants. 

Fast fashion was seen as contributing to climate change by 22% (n = 106) of Gen Z participants. The level of awareness here is also poor, with the World Economic Forum [40] identifying fashion to be the third largest supply chain that contributes 5% of global greenhouse gas emissions. Political inaction is acknowledged by 19% (n = 92) of Australia’s Gen Z which compares unfavourably with 49% of their American counterparts stating that political action on climate change should be prioritised [37]. A possible reason for this could be disenchantment with Australian politics and a lack of belief in political leadership [41].

A small section of the sample, namely 7% (n = 33) believe that the sun itself is also a major reason for climate change. This factor was not included in the original list but was added by the respective participants under the “other” option. The scientific evidence from the 6th Assessment Report of the Intergovernmental Panel on Climate Change (IPCC) shows unequivocally that human influence has warmed the climate at an unprecedented rate resulting in global surface temperature being 1.09 °C higher in 2011–2020 compared to 1850–1900 [42]. Natural factors, such as solar and volcanic activities, are estimated to have changed global surface temperature by −0.1 °C to 0.1 °C, making greenhouse gases the main driver of global warming [42]. Identifying the sun as a main contributor to climate change can be seen as either lack of knowledge or rejection of the scientific evidence. Previous studies have found that the number of climate deniers, that is people who reject global warming as human-made, in Australia, namely 8%, is much higher than the global average of 3% [43]. Although younger people are less likely to deny the connection between human activities and climate change, the share of those who blame the sun for climate change in this sample is considerably large. 

The findings of this study show that, irrespective of which factors Gen Z participants highlighted in their responses, they strongly believe that climate change is anthropogenic. Another Australian study reported similar results, where 96% of the participants believed climate change to be human made [44]. This is also consistent with the opinions of experts that there is a 97–98% consensus that humans are causing global warming [45]. 

Although all factors contributing, or otherwise, to global warming can be explored further, the focus of the remainder of this paper is on the nexus between dietary choices and climate change. This is done by using explicit quotes verbatim from the open-ended questions in the survey in order to convey directly the way young people feel about the issues. Also included are the year of birth, gender and place of residence of the participants when quoting them.

### 3.3. Human Food Choices and Climate Change according to Australia’s Gen Z

Gen Z’s involvement in strikes for climate and rallies globally and in Australia is undeniable, as this demographic cohort emerges as being the one responsible to deal with the mess past generations are leaving behind [31,44,46,47]. In this study, Gen Z sees climate change not only as a complex technological and scientific problem to solve, but also as a matter of changing the socio-political values of society as a whole and the will of policy leaders. For effective and lasting decision making, human civilisation needs a social tipping point that will overturn current thinking. This must happen before the tipping points in the planetary climate system are reached causing ecosystems to collapse. A 2022 survey by Deloitte of 14,808 Gen Z young people reached similar conclusions; 75% of the participants agree that the world is at a tipping point in responding to climate change and only a small percent, namely 11%, are of the opinion that their country’s government is highly committed to tackling this issue [35]. It may well be that this social turning point will come with Gen Z.

Surprisingly, not all of these young people are well-informed about the ecological footprint of people’s food choices. This was clearly visible in their responses. When asked directly whether food choices are a major contributor to climate change, Australia’s Gen Z was somehow divided. Two-thirds (66% or n = 316) of the participants replied negatively while 34% (n = 162) agreed. 

Table 2 presents some ‘for’ and ‘against’ explanations whether food choices contribute to climate change. In the ‘for’ argument, the participants explain that: “The production of beef alone results in a huge amount of greenhouse gasses being released”; “Human diet is also causing a lot to climate change” and “Meat and dairy account for around 15% of global greenhouse gas emissions”; while in the ‘against’ argument, they say: “Climate change has nothing to do with our human diet”; “Our food is not a problem” and “We have eaten meat all our life and we can’t blame it for causing climate change”. It is surprising, and to a certain degree alarming, that such a large share of this cohort, generally considered well-informed and interested in nutrition [48], lacks proper understanding of the climate change impacts of food choices. 

With much more participants of the opinion that human food choices, and particularly the consumption of animal-based products, are not causing climate change, Australia’s Gen Z appears to be not up to date with the scientific evidence in this field (e.g., [2,11,15]). Evidence from the UK shows that Gen Z is most likely to reduce meat consumption or eat no meat at all because of environmental concerns [14]. However, in Australia the study found Gen Z to be divided in its opinion about the environmental consequences of food choices and particularly meat production and consumption, to which they referred in the open-ended explanations. This may also be related to the claims Gen Z makes about being misunderstood [14], particularly as some continue to live at home, do not cook and have a common teenage diet reliant on burgers, chips and soft drinks [49]. Australia’s Gen Z typically under-consumes fruit and vegetables [49], similarly to the rest of Australia’s population, and in line with the other Australian age groups continues to increase its consumption of meat [3], albeit increasingly replacing beef with chicken, which has a smaller environmental footprint.

In the open-ended questions, the analysis found that, in general, Gen Z does not seem to engage with where their food comes from and does not pay much attention to labels. Opting to be vegan/vegetarian or buy organic produce may be more likely than among other generations but there seemed to be a trade-off between the price they were prepared to pay and the perceived health benefits. Animal welfare was mentioned by some of the participants but it was treated more as a social issue in terms of the conditions in which livestock animals are raised, rather than an environmental problem contributing to climate change, biodiversity loss, resource use and pollution. These young people seem to behave very similarly to their British counterparts who do not appear to be too worried about their food choices [14]. They simply take food for granted and rarely engage with deeper thoughts about its nature or origins [14,50]. Other studies, however, show a higher reaction to the environmental problems associated with food, with 35% of Gen Z stating that they have reduced, but not entirely ceased, their meat and fish consumption and another 19% reporting that they have become vegan or vegetarian [51].

Although the COVID-19 pandemic has influenced Gen Z in its appeal for more transparency, particularly in regard to how components are processed, the main focus remains on the link between food and mental health [51]. When it comes to food choices, Gen Z’s environmental values are not always in line with its actual consumption behaviour; for example, meat-based fast food and pre-packaged meals are frequently used in socialising [14]. 

Other studies show that Gen Z relies heavily on the content of social media about environmental issues [36], which is most likely to be the case regarding the nexus between food systems and climate change. Notwithstanding this, Gen Z wants transparency, evidence-based information and support from the food brands and governments promoting them [50]. Technology is seen as key to delivering food quality, variety and quantity, with less harmful impacts [14]. This brings to the fore the importance of and attitudes towards alternative proteins.

### 3.4. Alternative Proteins and Gen Z 

Alternative proteins, that is proteins obtained from sources other than livestock animals, are seen as an opportunity to improve food sustainability and security. They cover a wide range of options from plant-based analogues to familiar animal-sourced products, such as sausages, mince and dairy, to the use of insects and algae as foodstuffs, to cultured meat grown in bioreactors from animal cells [52]. Their proponents claim that there are environmental benefits from using alternative proteins compared to livestock products; they contribute towards improved nutrition, particularly given the limits on red meat consumption recommended in national dietary guidelines (including in Australia), and improve animal welfare by reducing the pressure to produce ever-increasing quantities of meat, dairy and other animal-sourced foodstuffs [53]. As of 2020, there were 16 alternative companies operating in Australia—13 using plant-based technologies, two working on cultured meat and one with algae [53]. There are also several research groups that are working on delivering alternative proteins, including at the Commonwealth Scientific and Industrial Research Organisation (CSIRO), university campuses and philanthropic organisations. Many plant-based alternatives are already available on the Australian market, either manufactured domestically or imported, with claims being made that they are, on the one hand, better for the environment and nutritionally and, on the other, similar in terms of taste, texture and other eating experience to conventional animal-based foods. 

Against this background, Australia’s Gen Z was highly divided in their attitudes expressed in the survey answers. The biggest separation in opinion was in relation to cultured meat. Only 28% of the Gen Z participants accepted cultured meat as a possible food option or “new form of protein”, “if we can master it”, to feed the growing human population. This acceptance share among Australian young people is much lower than that of more than half of consumers of all ages accepting cultured meat in Singapore (where this option has been legally sold in restaurants since 2020), USA [54] or Europe (France, Spain, the Netherlands and United Kingdom) [55].

The remaining 72% of Australia’s Gen Z participants stated that they were not ready to accept cultured meat. “Unnatural”, “abnormal”, “artificial” and “not normal” were the expressions most commonly used to describe this meat, echoing previous studies [16,49,56]. A range of reasons was put forward including personal doubts, related to the anticipated taste, disgust, concerns about health and safety, as well as societal considerations related to whether people need to accept at all the need to consume cultured meat and whether it is more sustainable. Socio-cultural perceptions were also used as a justification for the rejection of cultured meat, namely men need to eat meat (that is the link with masculinity) and Australian pride in producing high-quality livestock meat. Some also spoke about the ethics of using animal stem cells in the process of producing cultured meat without “the permission of the cow”, which could be “really unethical and painful”. Interestingly enough, for these people slaughtering livestock animals for meat seemed to be morally more acceptable. Finally, there were also those who described cultured meat as a conspiracy orchestrated by the rich and powerful against the average consumer and were determined not to be convinced to eat it.

Despite the lack of deeper understanding of the technologies and unfamiliarity with the ingredients used by the scientists and food producers to create novel alternative proteins [49], Gen Z instinctively feels that its relationship with these new foods is governed by the technological, textural and flavour complexities. However, it also accepts the magnitude of the problems associated with feeding a large global population in more sustainable ways. These resulted in 35% of the participants who reject cultured meat accepting plant-based substitutes and insects as more “natural” and “normal”. A further 9% approved only of insects which traditionally “are still eaten safely by many people in the world”. This statement is true as according to the United Nations Food and Agriculture Organization (FAO), two billion people currently consume insects as part of their diets [57].

Out of those who reject cultured meat, 17% disapprove of any alternatives because they are perceived as “chemically produced”, “heavily processed”, “Frankenstein food” and “not what our generation needs”. There were particularly concerns about the health and nutrition claims made by the producers, be it about plant-based alternatives, algae, insect-based or cultured meat. For the participating Gen Z representatives, understanding and critically scrutinising these products’ potential role in the present and future food systems and their capacity to help meet the nutritional needs of a growing global population are crucial. Gen Z wants concrete and reliable information rather than generic statements. These young people are far from seeing the alternative proteins revolutionising or disrupting the current food systems [13,54].

The last identified group of those who reject cultured meat are Gen Z participants who are ready to respond to the food challenges by increased consumption of real fruit and vegetables. Their share is relatively modest at 11%. These preferences for the traditional fruit and vegetables are seen as an environmentally conscious response [58].

Finally, the survey participants were asked what may persuade them to embrace alternative proteins, including cultured meat. The top reasons stated are broader sustainability concerns, including environmental impacts and contribution to climate change—most preferred answer (59%), resource depletion (44%) and health concerns (43%). Other reasons include population growth related food security (40%), animal welfare (24%) and fashion trends (22%). These results confirm that Gen Z is anxious about the natural environment and is also likely to adopt an eco-friendlier lifestyle for its food choices. In addition, with COVID-19 and the emergence of new zoonotic diseases, cultured meat and other alternative proteins may soon be perceived as safer, because they are produced in virus-free sterile environments controlled for human pathogens and where no antibiotics are used. This will decrease epidemiological risks and improve food security [59]. 

### 3.5. Protein Transition Insights

Globally and throughout the years, human population has witnessed major changes in the way food is produced and consumed [60]. Each generation has added its mark to human nutrition patterns and Gen Z is now at the brink of doing the same. However, this is happening in a historical period when climate change is looming as a major threat to human well-being and there have been responses from the food industry to provide new ways of feeding people. The evidence from the Australian Gen Z study gives some insights about the nexus between alternative proteins and climate change. 

Transition theories have been used in the past to explain changes in people’s behaviour in relation to food. This includes nutrition/protein transition theory which highlights the shift from plant- to animal-based foods initially and, in recent times, the reverse—from animal- to plant-based foods [61,62,63]. Aiking and de Boer [64] explain that alternative proteins would form part of the expected protein transition because of increased environmental and health awareness. Such a shift, however, is only at its very early stage. 

The findings from the study show that a large fraction of Australia’s Gen Z is not questioning its food choices and not embracing protein alternatives as a transition towards more sustainable plant-based options. Lack of knowledge about the real impact of animal-based foods on climate change is a contributing factor. How Gen Z can be influenced to reduce its consumption of animal-sourced foods and be motivated to incorporate more plant-based options in its diet requires additional research. 

Notwithstanding this, there are clear strategies that can impact such a transition. They should build on the sustainability of food production, resource use and health-related messages. Animal welfare and food-related fashion trends have a much more limited role to play in influencing Gen Z. Although not explored explicitly in this study, possible strategies can target decreasing the frequency of consumption of animal-based foods, mixing proteins of animal and plant origin in the same dish and reduction in portion sizes [65]. At the other end of the spectrum are strategies that promote increased consumption of traditional vegetables, fruits, legumes, tubers and nuts. Cultured meat does not appear attractive to the majority (namely 72%) of Australia’s Gen Z and is unlikely to play a major role in the nutrition transition. Food choices, however, are dynamic [66] and can shift with new social norms which respond to the climate change challenge and make alternative proteins more acceptable.

## 4. Conclusions

What is the main message to take away from this mixed-methods research? While Gen Z is ready to transform the world and global warming is high on its agenda, the nexus between climate change and food is yet to be properly understood by these Australian young people. This is a generation that is well-aware of the importance of transitioning away from fossil fuels and putting a stop to biodiversity loss and deforestation. Waste is similarly a concern for Gen Z and so are changing current consumption and lifestyle practices, making transport and industry more sustainable. Livestock products are seen as contributing to climate change by only 38% of Gen Z with the majority being of the opinion that “(a)griculture, petrol cars, carbon emissions, flying, mining are causing our climate to change, not what we eat”.

Given all available evidence about the impact of livestock on the fragile environment and atmosphere of this planet, the future of this new demographic generation is bleak, unless there is a change. There is not yet a clear acceptance of alternative proteins with only 28% of the Australian study sample agreeing that, if done properly, cultured meat could be a viable option. While Gen Z wants their food to be healthy, tasty and transparently produced, it may well be that for the time being more fruit and vegetables are a healthier and better sustainable option than animal-based products. The future will tell whether the novel alternative proteins will reach the plate of Gen Z, but what is needed for now is a better understanding of the nexus between climate change and food.

## Figures and Tables

**Table 1:** Demographic characteristics of the Sydney Gen Z sample.

Demographic Parameters	Category	Number and Share (%)	
Gender	Male	238	49.8%
	Female	240	50.2%
Age	18 years	51	10.6%
	19 years	52	10.9%
	20 years	53	11.1%
	21 years	54	11.3%
	22 years	53	11.1%
	23 years	55	11.5%
	24 years	53	11.1%
	25 years	54	11.3%
	26 years	53	11.1%
Family status	Single	409	85.6%
	De facto/Married	69	14.4%
Children	No children	441	92.3%
	With one child	24	5.0%
	With two children	13	2.7%
Household income	Under $50,000	86	18.0%
	$51,000 to $74,000	143	30.0%
	$75,000 to $100,000	159	33.2%
	$101,000 or more	90	18.8%
Employment	Full-time	198	41.4%
	Part-time	184	38.6%
	Studying	96	20.0%
Place of residence	Sydney	83	17.3%
	Melbourne	82	17.1%
	Brisbane	78	16.3%
	Perth	80	16.7%
	Canberra	77	16.1%
	Adelaide	78	16.3%
Total		478	100%

**Table 2:** Arguments for and against dietary choices’ contribution to climate change.

Pro	Against
“Absolutely responsible. Meat and dairy account for around 15% of global greenhouse gas emissions.” (born 1996, female, from Sydney)	“Climate change has nothing to do with our human diet. Only the animals suffer.” (born 1999, female, from Brisbane)
“To a certain degree the large amount of meat eaten by society which creates large amounts of cows needed to satisfy the consumption which is creating the large CO_2_ emissions.” (born 2000, male, from Perth)	“No way food choices to contribute to climate change. Only fossil fuels and pollution, cars etc. are causing climate change.” (born 1997, male, from Perth)
“Yes, I believe the clearing of land to house livestock in some parts of the world and the general increase in consumption of meat products are a cause of climate change.” (born 1998, female, from Melbourne)	“Not really. We have eaten meat all our life and we can’t blame it for causing climate change. Coal mining, gas burning, water pollution are major contributors.” (born 2001, female, from Adelaide)
“I do think we contribute to climate change as we consume lots of red meat [which] means that the cows etc. we eat for food release gas which causes more carbon dioxide in our atmosphere to start with, then add that to other issues and we have too much going on that our earth can’t handle.” (born 2000, female, from Sydney)	“I don’t think consumption of animal-based products and dairy are sources contributing to global warming. I think pollution, fossil fuel production are more responsible for the climate change we experience.” (born 2000, female, from Adelaide)
“Yes. Human diet is also causing a lot to climate change. Carbon output [is] increasing when we consume meat products and also in wastage of food.“ (born 2001, male, from Brisbane)	“Agriculture, petrol cars, carbon emissions, flying, mining are causing our climate to change, not what we eat.” (born 2000, male, from Melbourne)
“It’s true. The production of beef alone results in a huge amount of greenhouse gasses being released.” (born 2002, female, from Canberra)	“Growing world population, increased CO_2_ in atmosphere, poor environmental care by countries and communities is what makes global warming, not what we eat on a daily basis.” (born 1998, male, from Sydney)
“Yes, cattle farming increased greenhouse gas emissions and the government in Australia is not taking the necessary actions to make its fair share to solve the climate change problem.” (born 1997, female, from Sydney)	“No way. Excessive human burning of fossil fuels is far and away the main reason. These are causing long-term changes to the natural environment.” (born 1999, male, from Canberra)
“Yes, using fertilisers that contain nitrogen produces oxide emissions. Also, increasing livestock farming produces large amounts of methane from animals.” (born 1996, male, from Perth)	“The carbon emissions’ main contributors are mainly too many cars on the road, fossil fuel, waste. Our food has nothing to do with climate change. We should cut fuel consumption!” (born 1997, male, from Sydney)
“To some degree. Methane being produced by cattle doesn’t help the environment. Land clearing to produce food and palm oil plantations also contributes.” (born 1999, male, from Melbourne)	“Our food is not a problem. People produce so many things, factories are polluting the atmosphere.” (born 1998, female, from Melbourne)
“Yes—the food chain production, transportation, preparation, packaging and disposal all creates waste and uses power which increases carbon emissions and drives climate change.“ (born 1998, female, from Brisbane)	“It is not likely to have a link between food choices and climate change. Coal burning, plastic usage, cutting down forest, toxic gases…” (born 2001, male, from Perth)

## Data Availability

All data is available on request.
